# Development and validation of a real-time SYBR green PCR method for the detection and differentiation of *Babesia* and *Theileria* species (Apicomplexa: Piroplasmida) in hard ticks and cattle blood from Thailand

**DOI:** 10.1051/parasite/2025040

**Published:** 2025-08-25

**Authors:** May Thazin Kyaw, Penchom Janwan, Tongjit Thanchomnang, Rutchanee Rodpai, Ubon Tangkawanit, Patcharaporn Boonroumkaew, Lakkhana Sadaow, Pewpan M. Intapan, Wanchai Maleewong, Oranuch Sanpool

**Affiliations:** 1 Department of Parasitology, Faculty of Medicine, Khon Kaen University Khon Kaen 40002 Thailand; 2 Mekong Health Science Research Institute, Khon Kaen University Khon Kaen 40002 Thailand; 3 Medical Innovation and Technology Program, School of Allied Health Sciences, Walailak University Nakhon Si Thammarat Thailand; 4 Faculty of Medicine, Mahasarakham University Maha Sarakham 44000 Thailand; 5 Department of Medical Technology, Faculty of Allied Health Sciences, Nakhonratchasima College Nakhon Ratchasima 30000 Thailand; 6 Department of Entomology and Plant Pathology, Faculty of Agriculture, Khon Kaen University Khon Kaen 40002 Thailand

**Keywords:** *Babesia*, *Theileria*, Thailand, Cattle, Ticks-borne diseases, SYBR green real-time PCR

## Abstract

Tick-borne pathogens, particularly *Babesia* and *Theileria* species, are major threats to cattle production, causing economically significant diseases such as babesiosis and theileriosis. In this study, a real-time SYBR Green PCR assay was developed to detect *Babesia* and *Theileria* species in hard ticks (*N* = 65) and cattle blood samples (*N* = 143) from Thailand. Using primers targeting the mitochondrial cytochrome b gene for *Babesia* and the nuclear 18S rRNA gene for *Theileria*, the assay measured specific melting temperatures (Tm) for each species. The results showed distinct Tm values for *Babesia bigemina* (74.38 ± 0.04 °C), *Babesia bovis* (75.7 ± 0.06 °C), *Theileria orientalis* (74.61 ± 0.03 °C), *Theileria sinensis* (75.84 ± 0.03 °C), and *Theileria annulata* (74.06 ± 0.03 °C). The assay demonstrated high specificity, with a cutoff cycle threshold of < 35 cycles and a minimum detectable concentration of 10 copies/μL. Significant species differences in melting curves were confirmed using Tukey’s HSD test (*p* < 0.05). *Theileria orientalis* was detected in 8.4% of cattle blood samples, while *T. sinensis* was found in 25.9%, and *B. bigemina* in 0.7%. *Theileria orientalis* was also detected in 7.7% of tick samples, *T. sinensis* in 16.9%, and *B. bigemina* in 6.1%. The assay returned negative results for all non-target blood and tissue pathogens tested for specificity. This robust, high-throughput assay is highly effective for monitoring *Babesia* and *Theileria* infections, facilitating close surveillance and intervention efforts against tick-borne diseases in cattle.

## Abstract (fr)

Les agents pathogènes transmis par les tiques, en particulier les espèces de *Babesia* et de *Theileria*, constituent une menace majeure pour la production bovine, provoquant des maladies économiquement importantes telles que la babésiose et la theilériose. Dans cette étude, un test de PCR SYBR Green en temps réel a été développé pour détecter les espèces de *Babesia* et de *Theileria* dans des tiques dures (*N* = 65) et des échantillons de sang de bovins (*N* = 143) en Thaïlande. À l’aide d’amorces ciblant le gène du cytochrome b mitochondrial pour *Babesia* et le gène de l’ARNr 18S nucléaire pour *Theileria*, le test a mesuré les températures de fusion (Tm) spécifiques de chaque espèce. Les résultats ont montré des valeurs de Tm distinctes pour *Babesia bigemina* (74,38 ± 0,04 °C), *Babesia bovis* (75,7 ± 0,06 °C), *Theileria orientalis* (74,61 ± 0,03 °C), *Theileria sinensis* (75,84 ± 0,03 °C) et *Theileria annulata* (74,06 ± 0,03 °C). Le test a démontré une spécificité élevée, avec un seuil de cycle de coupure < 35 cycles et une concentration minimale détectable de 10 copies/μL. Les différences significatives entre les espèces dans les courbes de fusion ont été confirmées à l’aide du test HSD de Tukey (*p* < 0,05). *Theileria orientalis* a été détecté dans 8,4 % des échantillons de sang de bovins, tandis que *T. sinensis* a été trouvé dans 25,9 % et *B. bigemina* dans 0,7 %. *Theileria orientalis* a également été détectée dans 7,7 % des échantillons de tiques, *T. sinensis* dans 16,9 % et *B. bigemina* dans 6,1 %. Le test a donné des résultats négatifs pour tous les agents pathogènes sanguins et tissulaires non ciblés testés pour la spécificité. Ce test robuste et à haut débit est très efficace pour le suivi des infections à *Babesia* et *Theileria*, facilitant ainsi la surveillance étroite et les interventions contre les maladies transmises par les tiques chez les bovins.

## Introduction

Tick-borne diseases have a profound negative impact on the cattle industry, particularly in tropical and subtropical regions worldwide. In addition to reduced meat and milk production caused by morbidity and mortality, they can result in economic losses imposed by treatment and tick control measures, amounting to approximately US$13.9–18.7 billion globally per year [23, 24, 46]. In Thailand, a developing agricultural nation in Southeast Asia, the growth of the livestock industry has been limited by the high occurrence of tick-borne diseases, mainly babesiosis and theileriosis [1, 21]. These diseases are caused by the piroplasms *Babesia* and *Theileria*, respectively and are exclusively transmitted by blood-feeding ixodid hard ticks affecting cattle [46].

*Babesia bigemina* and *B. bovis* are the primary causative agents of bovine babesiosis in the tropics and subtropics [10]. They share a common tick vector, *Rhipicephalus (Boophilus) microplus,* which is the most widely distributed tick species in Thailand [21, 37]. After invading host erythrocytes, *Babesia* species multiply asexually and destroy host erythrocytes. Bovine theileriosis is caused by several *Theileria* spp., including *Theileria annulata* and *T. parva*, which cause malignant lymphoproliferative disorders, and *T. orientalis* and *T. sinensis*, which cause benign theileriosis [5, 7, 33]. These pathogens are transmitted by ticks of the genera *Rhipicephalus*, *Hyalomma*, and *Haemaphysalis* [9, 16]. However, *R. microplus* is the primary vector of bovine theileriosis in Thailand [37, 51], and benign *Theileria* spp. have been reported in both cattle and *R. microplus* ticks in the country [22, 37, 39, 44, 51]. *Theileria* species invade host leukocytes and erythrocytes, and multiply asexually. Babesiosis and theileriosis can cause high fever, hemolytic anemia, jaundice, abortion, and death in cattle depending on the infecting *Babesia* or *Theileria* species [5, 10, 35]. In *B. bovis* infection, neurological manifestations known as cerebral babesiosis can occur [48]. Cases of bovine babesiosis and benign theileriosis have been reported in different parts of Thailand [3, 14, 43, 45].

For the diagnosis of these diseases, the gold-standard method is the microscopic examination of Giemsa-stained blood smears, which is cost-effective but labor-intensive and not very sensitive [2, 8, 12]. Serological assays such as enzyme-linked immunosorbent assay (ELISA) have been developed, but they are usually time-consuming and can yield cross-reactions [1, 32]. In contrast, molecular detection methods provide high sensitivity and specificity [17, 26, 27]. Several molecular detection methods, such as conventional PCR (cPCR) and nested PCR, have been developed to diagnose bovine babesiosis and theileriosis in Thailand [3, 14, 22, 39]. However, these methods are low throughput, require manipulation of amplicons during gel fractionation, and have a high risk of PCR-product cross-contamination [4]. Quantitative PCR assays using specific probes have also been developed [11, 25]. They are highly specific and sensitive but are costly, making them unsuitable for detecting multiple species in a large number of samples. On the other hand, the SYBR Green real-time PCR method is much cheaper and adaptable for any target sequences. Although it is less specific than use of probes, melting-curve analysis can increase its specificity [6, 34]. Molecular detection methods have been developed in other countries; however, our study advances the field by establishing a high-throughput system for simultaneous screening of both cattle blood and tick vectors within a single laboratory setting.

In this study, we established a reliable, robust, and high-throughput SYBR Green real-time PCR assay targeting the 18S rRNA gene of *Theileria* spp. and mitochondrial cytochrome b (Cytb) gene of *Babesia* spp. in both cattle blood and tick vectors from southern and northern Thailand. This method, specifically optimized for local strains, ensures high sensitivity and specificity, simultaneously detects and distinguishes three *Theileria* spp. and two *Babesia* spp. based on their melting profiles (Tm), and the first to integrate host and vector surveillance into a standardized platform in Thailand, providing a model adaptable for large-scale surveillance in other endemic regions.

## Materials and methods

### Ethics approval

The cattle blood and tick samples used in this study were collected according to the Guidelines for Animal Experimentation of the National Research Council of Thailand and approved by the Animal Ethics Committee of the Faculty of Medicine, Khon Kaen University, Thailand (AMEDKKU 003/2020 and AMEDKKU 009/2022).

### Sample collection

Cattle blood samples (*N* = 143) were collected from apparently healthy beef cattle in northeastern provinces (Maha Sarakham, Khon Kaen, Nakhon Ratchasima, and Roi Et) and a southern province (Nakhon Si Thammarat) of Thailand. These samples were received from the biospecimen bank at the Department of Parasitology, Khon Kaen University. The tick samples (*N* = 65) were obtained from the biospecimen bank at the Department of Entomology and Plant Pathology, Khon Kaen University. They were collected from beef cattle in upper-northeastern Thailand (Provinces: Mukdahan, Bueng Kan, Nakhon Phanom, Khon Kaen, Loei, Sakon Nakhon, Roi-Et, Maha Sarakham, Nong Bua Lamphu, and Nong Khai) (Additional file 1: Tables S1, S2). Ticks were collected from different parts of cattle: neck, abdomen, ear, tail, udder-scrotum, and ano-vulva, carefully removed by forceps from the skin and transferred to 5 mL tubes containing 90% ethanol and then stored at −20 °C [50]. Morphological species identification was performed under a stereomicroscope using standard keys and guides [42, 49, 52].

### DNA extraction of cattle blood and tick samples

DNA was extracted from 200 μL of cattle blood using a NucleoSpin^®^ Blood Mini Kit (MACHEREY-NAGEL, Duren, Germany), according to the manufacturer’s instructions. Tick DNA was extracted using a NucleoSpin^®^ tissue kit (MACHEREY-NAGEL), according to the manufacturer’s instructions. Concentrations of genomic DNA (gDNA) in each extract were measured on a NanoDrop™ One Spectrophotometer (Thermo Fisher Scientific, Waltham, MA, USA) and the sample was stored at −20 °C until further use. The gDNAs of various blood and tissue pathogens stored at −70 °C in the Biobank of the Department of Parasitology, Faculty of Medicine, Khon Kaen University, including *Anaplasma* spp. (*N* = 2), *Babesia canis* (*N* = 2), *Ehrlichia* spp. (*N* = 2), *Hepatozoon* sp. (*N* = 1), *Plasmodium falciparum* (*N* = 1), *P. vivax* (*N* = 1), *Trypanosoma evansi* (*N* = 1), *Toxoplasma* (*N* = 1), *Sarcocystis hominis* (*N* = 1), *S. sinensis* (*N* = 1), and *S. cruzi* (*N* = 1) were used for specificity testing.

### Tick identification

Molecular confirmation of identity was performed according to a previous study [50]. A part of the *cox* 1 gene was amplified using conventional PCR with forward primer S0725 (F1) (5′–TAC TCT ACT AAT CAT AAA GAC ATT GG–3′) and reverse primer S0726 (R1) (5′–CCT CCT CCT GAA GGG TCA AAA AAT GA–3′). The amplified products were then directly sequenced in both directions using the same PCR primers on an Applied Biosystems 3730XL DNA Analyzer, provided by Bionix sequencing service (Seoul, South Korea). The resulting sequences were identified and compared with others in the GenBank database using the Basic Local Alignment Search Tool (BLAST) from the National Center for Biotechnology Information (U.S. National Library of Medicine, Bethesda, MD) (http://blast.ncbi.nlm.nih.gov/Blast.cgi). Out of 65 ticks, 60 were identified as *Rhipicephalus microplus* (OM760991–OM760995, OM761006, OM761015, OM761016, OM761020, OM761021, OM761024, OM761032, OM761033, OM761037–OM761059, OM761062, and OM761064) and 5 were *Haemaphysalis bispinosa* (OM760846–OM760849 and OM760853), provided in Tables S1, S2. Representative alignment comparison between sequences from this study and reference sequences is presented in Supplementary Fig. 1.

### Conventional PCR for screening of *Babesia* and *Theileria* infections

To obtain the positive samples for plasmid control construction, cPCR amplification was used to screen the cattle blood samples stored in biobank. This was performed by targeting a portion of the 18S rRNA gene for *Theileria* spp. [53] and of the mitochondrial cytochrome b (Cytb) gene for *B. bigemina* and *B. bovis* [13]. A PCR reaction mixture of total volume 50 μL was prepared containing 25 μL of 2× GoTaq^®^ Green Master Mix (Promega, Madison, WI, USA), 1 μL of each primer (0.2 μM), 8 μL of template, and 15 μL of double-distilled water. The PCR cycling program was as follows: initial denaturation at 94 °C for 5 min; 35 cycles of denaturation at 94 °C for 30 s, annealing at 48.5 °C for 30 s, extension at 72 °C for 30 s, followed by a final extension step at 72 °C for 7 min. Aliquots of 5 μL of each reaction were analyzed on agarose gel 2% (w/v) for *Babesia* spp., 1% (w/v) for *Theileria* spp.) in 0.5× TBE buffer. The PCR product for each strain was subjected to electrophoresis at 100 V/cm for 25 min, after which the gel was stained with RedSafe™ Nucleic Acid Staining Solution (iNtRON Biotechnology, Gyeonggi‑do, South Korea) and visualized using an Axygen^®^ Gel Documentation System-BL (Axygen, Corning, NY, USA). The PCR products were sequenced in both directions by ATCG Co., Ltd. Thailand, using the PCR primers as sequencing primers.

### Plasmid DNA construction

Amplicons obtained by cPCR from field-collected samples positive for *B. bigemina, T. orientalis*, and *T. sinensis* and synthetic DNA of *T. annulata* and *B. bovis* (Tsingke Biotech Co., Ltd., Beijing, China) were purified with a GenepHlow™ Gel Extraction Kit (Geneaid Biotech, New Taipei City, Taiwan), cloned into pGEM-T Easy vector (Promega) and transformed into *Escherichia coli* JM109 competent cells (Promega). Three colonies from each sample were collected, and the inserted gene was detected using the colony PCR technique with primers M13F (-20)/M13R (-24) [31]. PCR products were sent for sequencing by ATGC Co., Ltd., Thailand. Plasmid DNA was extracted using a NucleoSpin^®^ Plasmid EasyPure kit (MACHEREY-NAGEL). Plasmid DNA concentration was measured on a NanoDrop™ One Spectrophotometer (Thermo Fisher Scientific). Plasmids were serially diluted to 10^9^–1 copies/μL with sterile deionized water for use in developing the real-time PCR detection method.

### Real-time PCR (qPCR) analysis

Three separate pairs of specific primers were used for qPCR analysis. For detection of the two *Babesia* spp., primer pairs targeting mitochondrial cytochrome b (Cytb) were used: cbisg-1F: 5′–TGT TCC AGG AGA TGT TGA TTC–3′ and cbisg-2R: 5′–AGC ATG GAA ATA ACG AAG TGC–3′ for *B. bigemina*; cbosg-1: 5′–TGT TCC TGG AAG CGT TGA TTC–3′ and cbosg-2: 5′–AGC GTG AAA ATA ACG CAT TGC–3′ for *B. bovis* [13]. For detection of *Theileria* spp. (*T. annulata*, *T. orientalis*, and *T. sinensis*), a primer pair targeting the 18S rRNA gene (BovisT-5F: 5′–CGA GAC CTT AAC CTG CTA AAT AGG–3′ and BovisT-5R: 5′–CCC TCT AAG AAG CGA TAA CGG–3′) was used [53]. qPCR analysis was performed using a QuantStudio™ 6 Flex Real-time PCR system. The total volume of 25 μL of PCR reaction was prepared comprising 12.5 μL Maxima SYBR Green/ROX qPCR Master Mix (2×) (Thermo Fisher Scientific), 0.175 μL of each primer (0.07 μM), 2 μL of gDNA template, and 10.15 μL of nuclease-free water. The optimized qPCR reaction parameters were as follows: initial denaturation at 95 °C for 5 min, followed by 40 cycles of denaturation at 95 °C for 20 s, annealing temperature at 58 °C for 30 s, and extension at 72 °C for 45 s. Then, temperature was increased from 60 to 95 °C at 0.05 °C intervals, with a hold-time of 15 s at each step. Standard melting-curve analysis was performed at 95 °C for 15 s, 60 °C for 1 min, and 95 °C for 15 s for one cycle. The cutoff for the number of cycles (Ct) used in sample diagnosis was set to 35 to minimize the formation of primer-dimers. Under these conditions, the few non-specific products generated did not affect the interpretation of the amplification results.

The same cattle blood samples (*N* = 143), used in the developed assay, were amplified by cPCR followed by sequencing. Tick samples (*N* = 65) were amplified by cPCR and sequenced in the previous study [51]. Then, the results were compared with the developed qPCR assay.

### Data analysis

Data analysis and graphical representation of results were performed using numpy v2.1.1 [18], pandas v3.12 [30], matplotlib v3.9.2 package [19] in Python 3 to generate melting curves. For clustering analysis, we use the K-Means module from scikit-learn v1.5.2 [36] to separate different melting-temperature groups [28].

R software version 4.4.1 [40] and R Studio Desktop version 2024.04.2 + 764 [41] were used for the statistical analyses. Tukey’s HSD (honestly significant difference) test was used to analyze the melting temperatures, setting a statistical significance value of *p* < 0.05. The R package agricolae version 1.3–5 [15] was used to perform pairwise comparisons and one-way ANOVA.

## Results

### *Babesia-* and *Theileria-*positive control samples

The plasmid controls for all species were validated by sequence analysis and deposited in GenBank. The sequences of *B. bigemina* (PV751017), *B. bovis* (PV751018), *T. orientalis* (PV774666), *T. sinensis* (PV774665), and *T. annulata* (PV751019) showed 100% identity with reference sequences in GenBank, including *B. bigemina* (OP361312) from cattle in Brazil, *B. bovis* (CP125253) from the USA, *T. orientalis* (MH208642) from ticks in China, *T. sinensis* (MT271911) from Malaysia, and *T. annulata* (MT341858) from cattle in Italy (Supplementary Fig. 2).

### Sensitivity of plasmid control

**Figure 1:**
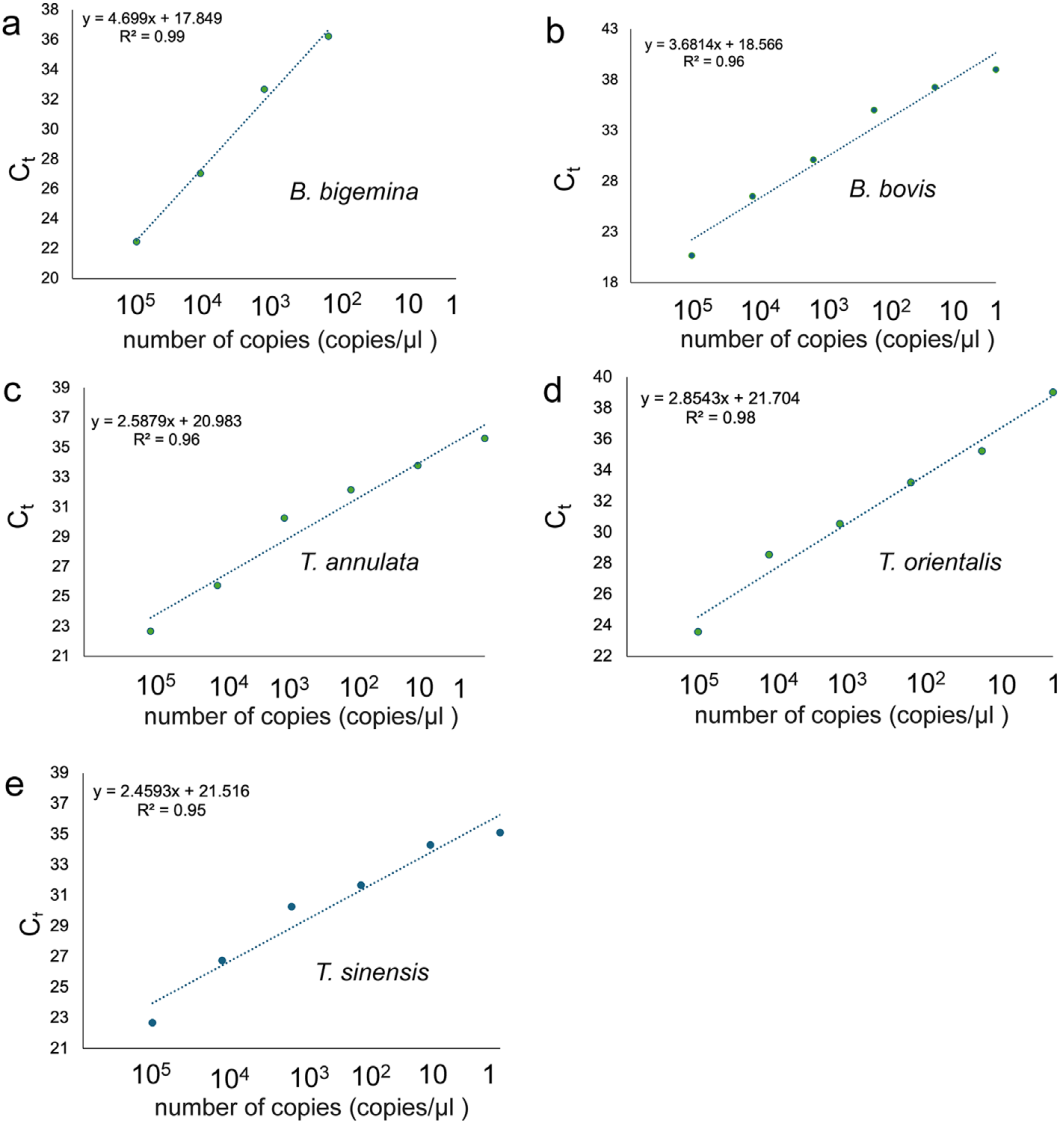
Standard curve of plasmid DNA dilutions (10^5^–1 copies/μL): a) *B. bigemina*, b) *B. bovis*, c) *T. annulata*, d) *T. orientalis*, and e) *T. sinensis*.

The cycle threshold (Ct) results from qPCR for each ten-fold plasmid dilution (ranging from 10^5^ copies to 1 copy; each time done in triplicate) were plotted to generate a standard curve. The detection limits for *B. bigemina* and *B. bovis* were 10^3^ copies/μL of plasmid DNA, *R*^2^ > 0.99 and *R*^2^ > 0.96, respectively (Figs. 1a–1b). *Theileria annulata* and *T. sinensis* qPCR sensitivity curves showed a lower limit of quantification of 10 copies/μL of plasmid DNA, with *R*^2^ > 0.96–0.95 (Figs. 1c, 1e). For *T. orientalis*, the qPCR sensitivity curves demonstrated a limit of quantification at 10^2^ copies/μL of plasmid DNA, with an *R*^2^ > 0.98 (Fig. 1d).

### Melting-curve analysis of qPCR assays

**Figure 2:**
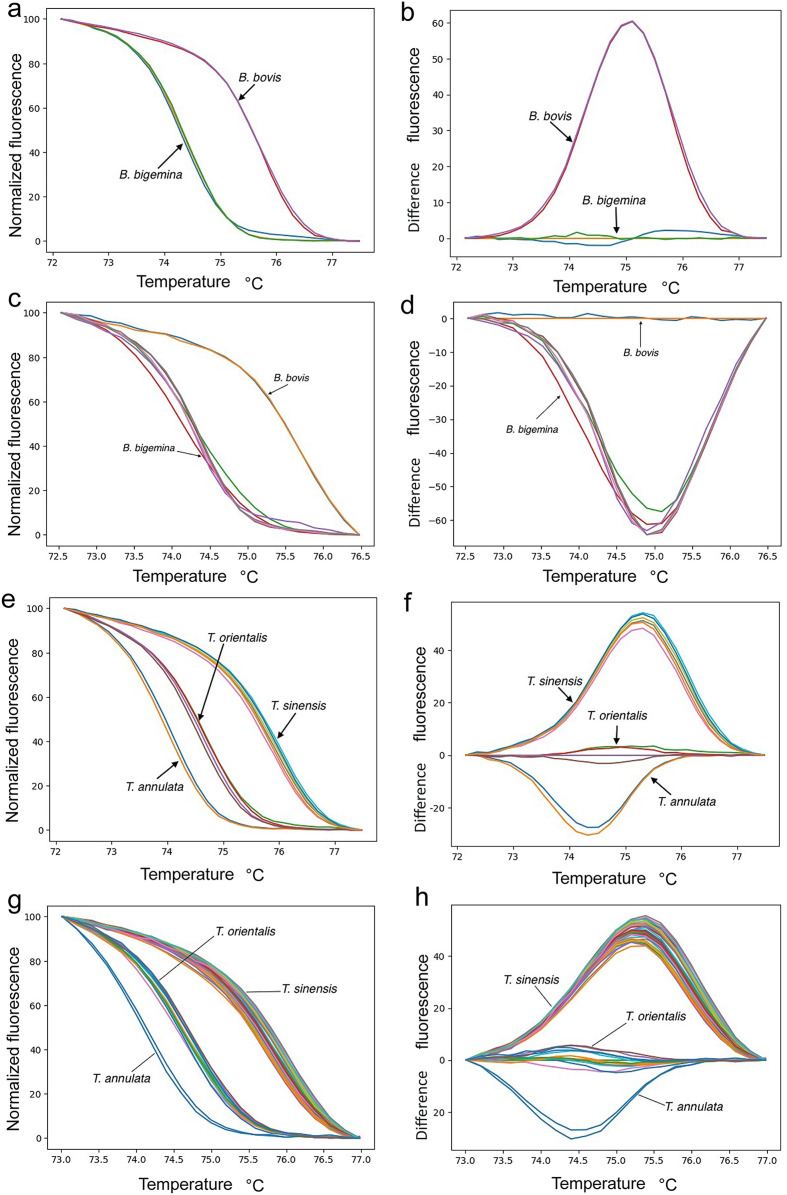
Melting-curve analysis of *Babesia* and *Theileria* species: a) Normalized melting curves, and b) Difference curves of *Babesia* spp. (Cytb gene) (plasmid controls), c) Normalized melting curves, and d) Difference curves of *Babesia* spp. (Cytb gene) (field samples compared with plasmid controls), e) Normalized melting curves, and f) Difference curves of *Theileria* spp. (18S rRNA gene) (plasmid controls), g) Normalized melting curves, and h) Difference curves of *Theileria* spp. (18S rRNA gene) (field samples compared with plasmid controls).

**Figure 3:**
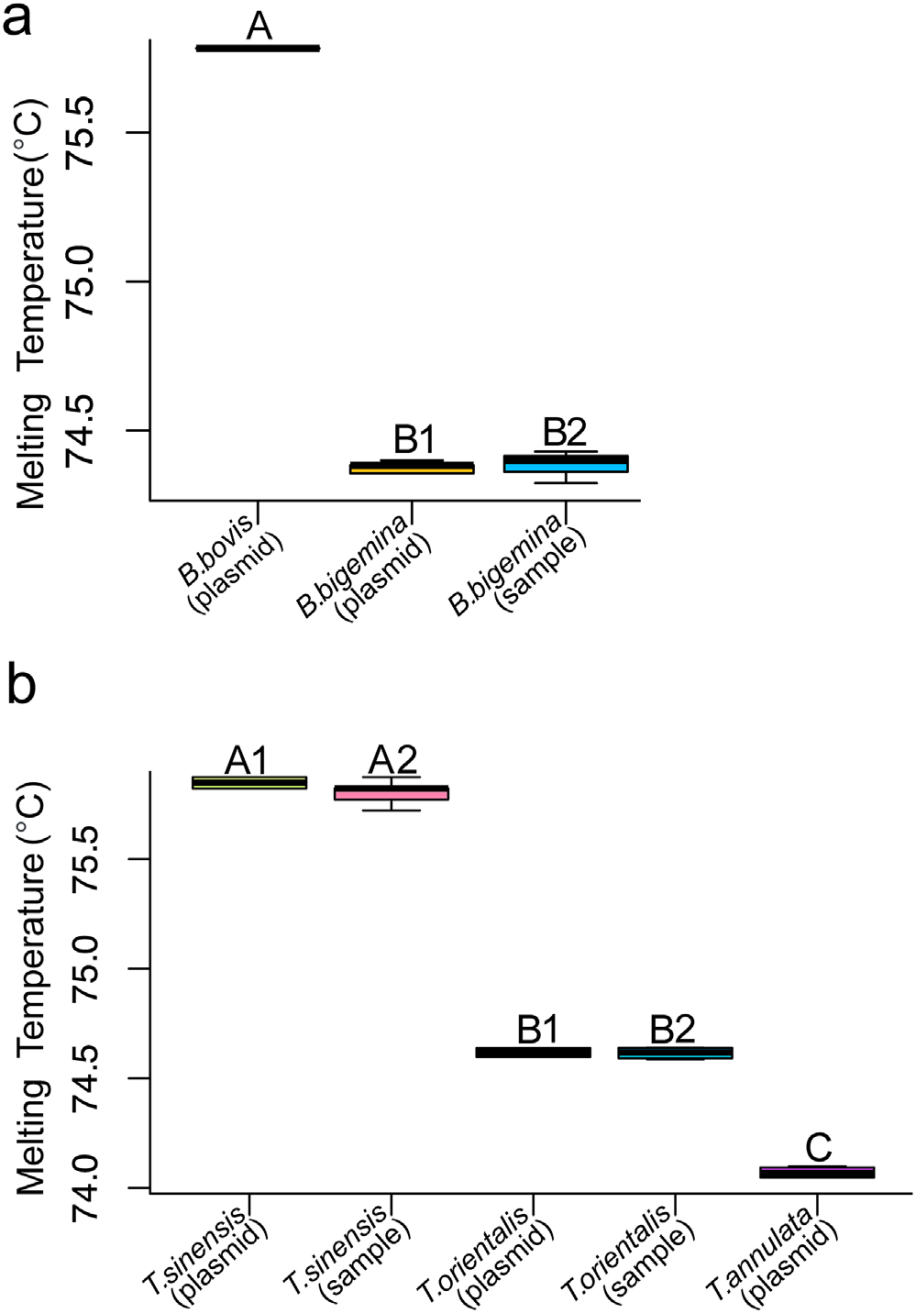
Melting temperatures boxplot of plasmid control (3 replicates) and samples of a) *Babesia* spp., and b) *Theileria* spp. Statistically significant differences (Tukey’s HSD test, *p < 0.05*) are indicated in the figure with different letters; boxplot with the same letter indicates no statistical difference. Figure 3a), A: plasmid controls for *B. bovis*, B1: plasmid controls for *B. bigemina*, B2: positive samples for *B. bigemina*. Figure 3b), A1: plasmid control for *T. sinensis*, A2: positive samples for *T. sinensis*, B1: plasmid controls for *T. orientalis*, B2: positive samples for *T. orientalis*, C: plasmid control for *T. annulata.*

Melting-curve analysis with a cycle cutoff of <35 cycles clearly distinguished the plasmid controls for *B. bigemina*, *B. bovis*, *T. annulata*, *T. orientalis*, and *T. sinensis*, with each species showing a distinct melting curve (Figs. 2a, 2b, 2e, 2f). Pairwise comparisons using Tukey’s HSD test revealed significant differences in melting temperatures among all species (*p* < 0.05). The melting temperatures were as follows: *B. bigemina* 74.38 ± 0.04 °C, *B. bovis* 75.7 ± 0.06 °C (Fig. 3a), *T. annulata* 74.06 ± 0.03 °C, *T. orientalis* 74.61 ± 0.03 °C, and *T. sinensis* 75.84 ± 0.03 °C (Fig. 3b).

### Evaluation of the qPCR assay using plasmid controls and field samples

Melting-curve analysis of the cattle blood samples compared with plasmid controls revealed *Theileria orientalis* in 12 out of 143 (8.4%), *T. sinensis* in 37 out of 143 (25.9%), and *Babesia bigemina* in 1 out of 143 (0.7%). In the tick samples, *Theileria orientalis* was found in 5 out of 65 specimens (7.7%), *T. sinensis* in 11 specimens (16.9%), and *B. bigemina* in 4 specimens (6.1%), from *R. microplus* only (Additional files 1 and 2: Tables S1, S2). The melting curves for each species were clearly distinguishable (Figs. 2c, 2d, 2g, 2h). A comparison of the mean melting temperature for each species using Tukey’s HSD tests indicated statistically significant differences (Fig. 3) (*p* < 0.05). Neither *B. bovis* nor *T. annulata* was detected in any of the samples. Additionally, none of the other biobank samples tested for specificity (*Anaplasma* spp., *B. canis*, *Ehrlichia* spp., *Hepatozoon* sp., *P. falciparum*, *P. vivax*, *T. evansi*, *Toxoplasma*, *S. hominis*, *S. sinensis*, and *S. cruzi*) yielded positive results, as all showed Ct values above the detection cutoff (>35).

cPCR amplification of cattle blood samples (*N* = 143) showed 4 samples positive for *B. bigemina*, and sequencing confirmed the identity (not submitted to GenBank due to short sequence length), 3 samples for *T. orientalis* (PV592335 and PV592336), and 17 samples for *T. sinensis* (PV592339–PV592343) (Supplementary Fig. 2). Those of tick samples were 4 samples positive for *B. bigemina*, 5 samples positive for *T. orientalis* (Accession numbers: PP30060–PP30063) and 12 samples for *T. sinensis*, all positive sequences were submitted to the GenBank database (Accession numbers: PP188642–PP188663) [51].

## Discussion

This study successfully developed and validated a high-throughput qPCR assay for the detection and differentiation of *Babesia bigemina*, *B. bovis*, *Theileria orientalis*, *T. sinensis*, and *T. annulata* in both cattle blood and tick vectors. Compared to traditional microscopy and serological methods, this qPCR assay offers superior sensitivity, allowing early detection and differentiation of multiple pathogens in co-infected cases [20, 21, 29, 47]. Its high-throughput nature makes it a valuable tool for large-scale epidemiological surveillance and disease control [43]. The robustness of the assay was evident through distinct melting curves, with statistically significant differences in melting temperatures between species. This highlights the assay’s specificity in differentiating closely related species of *Babesia* and *Theileria*, which is critical for accurate diagnosis and effective disease management.

Field samples revealed a high prevalence of *T. sinensis* and *T. orientalis* in tick vectors, consistent with reports of benign theileriosis in Southeast Asia [5, 35]. The detection of *B. bigemina* in cattle blood at lower prevalence aligns with previous studies documenting the endemicity of bovine babesiosis in Thailand [43, 45]. Since most of the tick vectors in this study were *R. microplus*, this also corroborates previous studies on the abundance of this species in Thailand, and its role in the possible transmission of *B. bigemina*, *B. bovis*, *T. orientalis*, and *T*. *sinensis* [21, 37]. Neither *Babesia* nor *Theileria* spp. were detected in *H. bispinosa* which is also a cattle tick, but not a known vector for these species. However, only 6 ticks of the species were tested. The absence of *B. bovis* and *T. annulata* in the field samples may reflect regional differences in tick vector populations or could be attributed to limited sample sizes. Similar studies have suggested that such variations may result from environmental factors, vector competence, and host availability, which influence the distribution of these pathogens [30, 38]. Despite its numerous advantages, the qPCR assay has some limitations. The main limitation is the reliance on high-quality DNA; low-quality samples may result in false negatives, highlighting the importance of optimized sample collection and storage methods. This assay showed negative for samples that were positive for *B. bigemina* by cPCR and sequencing*.* This can be due to extremely low concentration of parasites. The implications of this study are significant for disease management and control strategies in cattle populations. The ability to accurately distinguish between *Babesia* and *Theileria* species enables targeted treatment approaches, reducing the misuse of chemotherapeutic agents and the emergence of drug resistance​ [35]. Furthermore, the assay can serve as a valuable epidemiological tool to monitor the spread of tick-borne pathogens, aiding in the development of effective vector control programs [43]. The presence of genetically different strains of *Theileria orientalis* and *Babesia bovis* in different regions suggests that future diagnostic tools should incorporate broader strain-specific primers to enhance detection accuracy [45]. Moreover, the findings emphasize the importance of region-specific surveillance to mitigate disease outbreaks and improve livestock health management strategies [37].

## Conclusions

This study successfully developed and validated a high-throughput qPCR assay for the detection and differentiation of *B. bigemina*, *B. bovis*, *T. orientalis*, *T. sinensis*, and *T. annulata* in cattle blood and tick vectors. The assay demonstrated high specificity and robustness, with clear differentiation between closely related species based on melting curves and statistically significant temperature differences. Field sample analyses indicated a high prevalence of *T. sinensis* and *T. orientalis* in the tick vector, *R. microplus*, highlighting its role in transmission of bovine theileriosis – in addition to bovine babesiosis – in Thailand. Cattle blood samples showed a lower prevalence of *B. bigemina*, consistent with previous reports from Southeast Asia. The absence of *B. bovis* and *T. annulata* may reflect regional differences or sample limitations. Overall, the real-time SYBR Green PCR assay developed here is a valuable molecular tool for early and accurate diagnosis of tick-borne protozoan infections, enhancing efforts in disease management and control. Further research into the geographic distribution of tick vectors and pathogens is essential for better-targeted interventions.

## Supplementary material

*Supplementary Figure 1*: Homology comparisons of reference sequences and sequences submitted in this study for tick species identification. OR545517: reference sequence of *R. microplus*, OR335052: reference sequence of *H. bispinosa*, Representative sequences from each province (KK: Khon Kaen, BK: Bueng Kan, SK: Sakon Nakhon, NP: Nakhon Phanom, LI: Loei, RE: Roi Et, and MS: Maha Sarakham), were aligned. *Supplementary Figure 2*: Comparison of plasmid control sequences with GenBank references. a) Plasmid controls: *B. bigemina* (PV751017) and *B. bovis* (PV751018), reference sequences: *B. bigemina* (OP361312) and *B. bovis* (CP125253), b) Plasmid controls: *T. annulata* (PV751019), *T. orientalis* (PV774666), and *T. sinensis* (PV774665), references sequences: *T. annulata* (MT341858), *T. orientalis* (MH208642), and *T. sinensis* (MT271911). *Table S1*: Real-Time PCR results of *Theileria* detection. *Table S2*: Real-Time PCR results of *Babesia* detection. The supplementary material for this article can be found at https://www.parasite-journal.org/10.1051/parasite/2025040/olm.
