# Supplementary material for: Development and validation of a real-time SYBR green PCR method for the detection and differentiation of Babesia and Theileria species (Apicomplexa: Piroplasmida) in hard ticks and cattle blood from Thailand
Source: Parasite. 2025 Aug 25;32:54. doi: 10.1051/parasite/2025040 (PMC12380414; doi:10.1051/parasite/2025040)
Supplement: Supplementary file 1 — Supplementary Figure 1: Homology comparisons of reference sequences and sequences submitted in this study for tick species identification. OR545517: reference sequence of R. microplus, OR335052: reference sequence of H. bispinosa, Representative sequences from each province (KK: Khon Kaen, BK: Bueng Kan, SK: Sakon Nakhon, NP: Nakhon Phanom, LI: Loei, RE: Roi Et, and MS: Maha Sarakham), were aligned. Supplementary Figure 2: Comparison of plasmid control sequences with GenBank references. a) Plasmid controls: B. bigemina (PV751017) and B. bovis (PV751018), reference sequences: B. bigemina (OP361312) and B. bovis (CP125253), b) Plasmid controls: T. annulata (PV751019), T. orientalis (PV774666), and T. sinensis (PV774665), references sequences: T. annulata (MT341858), T. orientalis (MH208642), and T. sinensis (MT271911). Table S1: Real-Time PCR results of Theileria detection. Table S2: Real-Time PCR results of Babesia detection. [file parasite-32-54-s1.zip › Table S1 clean.docx]

**Table S1.** Real-Time PCR results of *Theileria* detection.

| Sample name | Sample type | Location | Result (cPCR) | Result (qPCR) C_t_ (cutoff<35) | T_m_ | Remark |
| --- | --- | --- | --- | --- | --- | --- |
| MKM194 | cow blood | Maha Sarakham | Negative | Undetected | 74.737 |  |
| MKM195 | cow blood | Maha Sarakham | Negative | Undetected | 61.114 |  |
| MKM196 | cow blood | Maha Sarakham | Negative | 36.988 | 74.737 |  |
| MKM197 | cow blood | Maha Sarakham | Negative* | 30.722 | 74.638 |  |
| MKM198 | cow blood | Maha Sarakham | Negative | Undetected | 76.02 |  |
| MKM199 | cow blood | Maha Sarakham | Negative | Undetected | 74.835 |  |
| MKM200 | cow blood | Maha Sarakham | Negative | Undetected | 74.539 |  |
| MKM201 | cow blood | Maha Sarakham | Negative | Undetected | 76.02 |  |
| MKM202 | cow blood | Maha Sarakham | Negative | Undetected | 75.033 |  |
| MKM203 | cow blood | Maha Sarakham | Negative | Undetected | 75.724 |  |
| MKM204 | cow blood | Maha Sarakham | Negative | Undetected | 74.934 |  |
| MKM205 | cow blood | Maha Sarakham | Negative | Undetected | 74.835 |  |
| MKM206 | cow blood | Maha Sarakham | Negative | Undetected | 63.286 |  |
| MKM207 | cow blood | Maha Sarakham | Negative | Undetected | 66.05 |  |
| MKM208 | cow blood | Maha Sarakham | Negative | Undetected | 74.835 |  |
| MKM209 | cow blood | Maha Sarakham | Negative | Undetected | 62.694 |  |
| MKM210 | cow blood | Maha Sarakham | Negative | Undetected | 75.921 |  |
| MKM211 | cow blood | Maha Sarakham | Negative | Undetected | 75.23 |  |
| MKM212 | cow blood | Maha Sarakham | Negative | Undetected | 61.904 |  |
| MKM213 | cow blood | Maha Sarakham | Negative | Undetected | 76.02 |  |
| MKM214 | cow blood | Maha Sarakham | Negative | Undetected | 75.033 |  |
| MKM215 | cow blood | Maha Sarakham | Negative | 39.453 | 75.625 |  |
| MKM216 | cow blood | Maha Sarakham | Negative | Undetected | 75.921 |  |
| MKM217 | cow blood | Maha Sarakham | Negative | Undetected | 62.003 |  |
| MKM218 | cow blood | Maha Sarakham | Negative | Undetected | 75.23 |  |
| MKM219 | cow blood | Maha Sarakham | Negative | Undetected | 75.823 |  |
| MKM220 | cow blood | Maha Sarakham | Negative | Undetected | 75.329 |  |
| MKM221 | cow blood | Maha Sarakham | Negative | Undetected | 64.076 |  |
| MKM222 | cow blood | Maha Sarakham | Negative | 39.547 | 74.835 |  |
| MKM223 | cow blood | Maha Sarakham | Negative | 38.051 | 75.724 |  |
| MKM224 | cow blood | Maha Sarakham | Negative | 38.656 | 75.033 |  |
| MKM225 | cow blood | Maha Sarakham | Negative | Undetected | 74.539 |  |
| KKN 2 | cow blood | Khon Kean | Negative | 35.57 | 74.785 |  |
| KKN 3 | cow blood | Khon Kean | Negative | Undetected | 76.073 |  |
| KKN 4 | cow blood | Khon Kean | Negative | Undetected | 76.172 |  |
| KKN 5 | cow blood | Khon Kean | Negative | 37.555 | 75.776 |  |
| KKN 6 | cow blood | Khon Kean | Negative | 37.395 | 75.875 |  |
| KKN 7 | cow blood | Khon Kean | Negative | Undetected | 74.488 |  |
| KKN 8 | cow blood | Khon Kean | Negative | 36.957 | 75.875 |  |
| KKN 9 | cow blood | Khon Kean | Negative | 36.126 | 75.875 |  |
| KKN 10 | cow blood | Khon Kean | Negative | 39.988 | 75.776 |  |
| KKN 11 | cow blood | Khon Kean | Negative | 38.894 | 74.884 |  |
| KKN 12 | cow blood | Khon Kean | Negative | 36.653 | 75.577 |  |
| KKN 13 | cow blood | Khon Kean | Negative | 38.159 | 75.875 |  |
| KKN 14 | cow blood | Khon Kean | Negative | Undetected | 76.172 |  |
| KKN 15 | cow blood | Khon Kean | Negative | 37.375 | 75.875 |  |
| KKN 16 | cow blood | Khon Kean | Negative | 37.213 | 74.785 |  |
| KKN 17 | cow blood | Khon Kean | Negative | Undetected | 76.073 |  |
| KKN 18 | cow blood | Khon Kean | Negative | 38.547 | 74.884 |  |
| KKN 19 | cow blood | Khon Kean | Negative | Undetected | 75.478 |  |
| KKN 20 | cow blood | Khon Kean | Negative | 35.282 | 75.577 |  |
| KKN 21 | cow blood | Khon Kean | Negative | Undetected | 75.974 |  |
| KKN 22 | cow blood | Khon Kean | Negative | 39.132 | 75.28 |  |
| KKN 23 | cow blood | Khon Kean | Negative | 39.249 | 75.478 |  |
| NMA 8 | cow blood | Nakhon Ratchasima | *T. sinensis*  (PV592339) ** | 27.712 | 75.716 |  |
| NMA 83 | cow blood | Nakhon Ratchasima | *T. orientalis*  (PV592336) ** | 31.1 | 74.79 |  |
| NMA 88 | cow blood | Nakhon Ratchasima | *T. orientalis*  (PV592336) ** | 35.41 | 74.719 |  |
| NMA128 | cow blood | Nakhon Ratchasima | Negative | Undetected | 74.781 |  |
| NMA129 | cow blood | Nakhon Ratchasima | Negative* | 28.417 | 74.682 |  |
| NMA130 | cow blood | Nakhon Ratchasima | Negative | Undetected | 74.682 |  |
| NMA131 | cow blood | Nakhon Ratchasima | Negative* | 27.032 | 74.682 |  |
| NMA132 | cow blood | Nakhon Ratchasima | Negative* | 28.132 | 74.682 |  |
| NMA133 | cow blood | Nakhon Ratchasima | Negative | Undetected | 74.385 |  |
| NMA134 | cow blood | Nakhon Ratchasima | Negative | Undetected | 74.781 |  |
| NMA135 | cow blood | Nakhon Ratchasima | Negative* | 24.451 | 74.583 |  |
| NMA136 | cow blood | Nakhon Ratchasima | Negative* | 29.74 | 74.682 |  |
| NMA137 | cow blood | Nakhon Ratchasima | Negative* | 28.122 | 74.88 |  |
| NMA139 | cow blood | Nakhon Ratchasima | Negative* | 33.3 | 75.474 |  |
| NMA138 | cow blood | Nakhon Ratchasima | Negative | 39.763 | 75.871 |  |
| NMA141 | cow blood | Nakhon Ratchasima | Negative | Undetected | 74.781 |  |
| NMA140 | cow blood | Nakhon Ratchasima | Negative* | 33.194 | 75.772 |  |
| NMA143 | cow blood | Nakhon Ratchasima | Negative | Undetected | 75.97 |  |
| NMA142 | cow blood | Nakhon Ratchasima | Negative | Undetected | 74.88 |  |
| NMA146 | cow blood | Nakhon Ratchasima | Negative* | 28.414 | 74.682 |  |
| NMA144 | cow blood | Nakhon Ratchasima | Negative* | 34.371 | 74.682 |  |
| NMA147 | cow blood | Nakhon Ratchasima | Negative | Undetected | 61.711 |  |
| NMA148 | cow blood | Nakhon Ratchasima | Negative | Undetected | 61.117 |  |
| NMA149 | cow blood | Nakhon Ratchasima | Negative | 38.275 | 74.781 |  |
| NMA150 | cow blood | Nakhon Ratchasima | Negative | Undetected | 61.315 |  |
| NMA151 | cow blood | Nakhon Ratchasima | Negative | Undetected | 60.721 |  |
| NMA152 | cow blood | Nakhon Ratchasima | Negative* | 27.72 | 74.781 |  |
| NMA153 | cow blood | Nakhon Ratchasima | Negative | Undetected | 75.871 |  |
| NMA154 | cow blood | Nakhon Ratchasima | Negative | 39.524 | 74.88 |  |
| NMA155 | cow blood | Nakhon Ratchasima | Negative* | 28.152 | 74.781 |  |
| NMA156 | cow blood | Nakhon Ratchasima | Negative | Undetected | 62.305 |  |
| NMA157 | cow blood | Nakhon Ratchasima | Negative* | 26.745 | 74.781 |  |
| NMA158 | cow blood | Nakhon Ratchasima | Negative | Undetected | 74.88 |  |
| NMA159 | cow blood | Nakhon Ratchasima | Negative | Undetected | 74.88 |  |
| NMA160 | cow blood | Nakhon Ratchasima | Negative | Undetected | 75.078 |  |
| NMA174 | cow blood | Nakhon Ratchasima | *T. orientalis*  (PV592335) ** | 25.031 | 74.631 |  |
| NST1 | cow blood | Nakhon Si Thammarat | *T. sinensis* (PV592343) **** | 27.651 | 75.702 | Mix *B. bigemina* |
| NST2 | cow blood | Nakhon Si Thammarat | *T. sinensis*  (PV592343) ** | 25.998 | 75.725 |  |
| NST3 | cow blood | Nakhon Si Thammarat | Negative | Undetected | 75.078 |  |
| NST4 | cow blood | Nakhon Si Thammarat | *T. sinensis*  (PV592339) ** | 24.068 | 75.725 |  |
| NST5 | cow blood | Nakhon Si Thammarat | *T. sinensis*  (PV592339) ** | 30.103 | 75.824 |  |
| NST6 | cow blood | Nakhon Si Thammarat | *T. sinensis*  (PV592339) ** | 28.338 | 75.824 |  |
| NST7 | cow blood | Nakhon Si Thammarat | *T. sinensis*  (PV592340) ** | 30.482 | 75.824 |  |
| NST8 | cow blood | Nakhon Si Thammarat | *T. sinensis*  (PV592339) ** | 28.577 | 75.725 |  |
| NST9 | cow blood | Nakhon Si Thammarat | *T. sinensis*  (PV592339) ** | 29.592 | 75.725 |  |
| NST10 | cow blood | Nakhon Si Thammarat | Negative* | 34.121 | 75.725 |  |
| NST11 | cow blood | Nakhon Si Thammarat | *T. sinensis*  (PV592339) ** | 31.13 | 75.824 |  |
| NST12 | cow blood | Nakhon Si Thammarat | Negative* | 25.952 | 75.725 |  |
| NST14 | cow blood | Nakhon Si Thammarat | *T. sinensis*  (PV592341) **** | 30.171 | 75.824 |  |
| NST15 | cow blood | Nakhon Si Thammarat | *T. sinensis*  (PV592339) ** | 26.883 | 75.824 |  |
| NST16 | cow blood | Nakhon Si Thammarat | *T. sinensis*  (PV592339) ** | 26.932 | 75.824 | Mix *B. bigemina* |
| NST17 | cow blood | Nakhon Si Thammarat | *T. sinensis*  (PV592339) ** | 26.578 | 75.725 |  |
| NST19 | cow blood | Nakhon Si Thammarat | *T. sinensis*  (PV592342) **** | 27.162 | 75.824 |  |
| NST20 | cow blood | Nakhon Si Thammarat | *T. sinensis*  (PV592339) ** | 27.152 | 75.824 |  |
| NST21 | cow blood | Nakhon Si Thammarat | Negative* | 26.228 | 75.824 |  |
| NST22 | cow blood | Nakhon Si Thammarat | Negative* | 29.659 | 75.824 |  |
| NST23 | cow blood | Nakhon Si Thammarat | Negative* | 26.932 | 75.824 |  |
| NST24 | cow blood | Nakhon Si Thammarat | Negative* | 27.851 | 75.725 |  |
| NST25 | cow blood | Nakhon Si Thammarat | Negative* | 29.972 | 75.824 |  |
| NST26 | cow blood | Nakhon Si Thammarat | Negative* | 29.842 | 75.923 |  |
| NST27 | cow blood | Nakhon Si Thammarat | Negative* | 29.366 | 75.923 |  |
| NST28 | cow blood | Nakhon Si Thammarat | Negative* | 30.973 | 75.824 |  |
| NST29 | cow blood | Nakhon Si Thammarat | Negative* | 29.462 | 75.899 |  |
| NST30 | cow blood | Nakhon Si Thammarat | *T. sinensis*  (PV592339) ** | 27.672 | 75.8 | Mix *B. bigemina* |
| NST31 | cow blood | Nakhon Si Thammarat | Negative* | 28.412 | 75.824 |  |
| NST32 | cow blood | Nakhon Si Thammarat | Negative* | 27.764 | 74.738 |  |
| NST33 | cow blood | Nakhon Si Thammarat | Negative* | 28.473 | 75.923 |  |
| NST34 | cow blood | Nakhon Si Thammarat | Negative* | 30.313 | 75.824 |  |
| NST35 | cow blood | Nakhon Si Thammarat | Negative* | 28.366 | 75.824 |  |
| NST36 | cow blood | Nakhon Si Thammarat | Negative* | 23.159 | 61.415 |  |
| NST37 | cow blood | Nakhon Si Thammarat | Negative | 35.232 | 75.923 |  |
| NST38 | cow blood | Nakhon Si Thammarat | Negative* | 25.982 | 74.936 |  |
| NST39 | cow blood | Nakhon Si Thammarat | Negative* | 27.093 | 75.923 |  |
| NST40 | cow blood | Nakhon Si Thammarat | Negative* | 28.307 | 74.738 |  |
| NST41 | cow blood | Nakhon Si Thammarat | Negative* | 24.669 | 74.541 |  |
| NST42 | cow blood | Nakhon Si Thammarat | Negative* | 26.297 | 75.923 |  |
| NST43 | cow blood | Nakhon Si Thammarat | Negative* | 30.435 | 75.923 |  |
| NST44 | cow blood | Nakhon Si Thammarat | Negative* | 25.268 | 75.8 |  |
| RET1 | cow blood | Roi-Et | Negative* | 24.686 | 74.797 |  |
| RET2 | cow blood | Roi-Et | Negative* | 25.811 | 74.698 |  |
| RET3 | cow blood | Roi-Et | Negative* | 22.076 | 74.599 |  |
| RET4 | cow blood | Roi-Et | Negative* | 25.203 | 74.5 |  |
| RET5 | cow blood | Roi-Et | Negative* | 25.069 | 74.599 |  |
| RET6 | cow blood | Roi-Et | Negative* | 26.157 | 74.698 |  |
| RET7 | cow blood | Roi-Et | Negative* | 28.747 | 74.797 |  |
| RET8 | cow blood | Roi-Et | Negative* | 28.475 | 74.797 |  |
| RET9 | cow blood | Roi-Et | Negative* | 27.711 | 74.698 |  |
| RET10 | cow blood | Roi-Et | Negative* | 27.48 | 74.797 |  |
| RET11 | cow blood | Roi-Et | Negative* | 27.723 | 74.587 |  |
| Tick 22 | Tick  (*R.* *microplus -* OM760994)** [50] | Mukdahan | *Anaplasma* | Undetected | 85.502 |  |
| Tick 67 | Tick  (*R. microplus -* OM761040)** [50] | Bueng Kan | *Anaplasma* | Undetected | 85.205 |  |
| Tick 81 | Tick  (*R. microplus -* OM761006)** [50] | Nakhon Phanom | *T. orientalis*  (PP330060) ** [51] | Undetected | 85.602 |  |
| Tick 92 | Tick  (*R.* *microplus -* OM760991)** [50] | Khon Kean | Negative | Undetected | 84.805 |  |
| Tick 93 | Tick  (*R. microplus-* OM761020)** [50] | Loei | Negative | 38.403 | 74.442 |  |
| Tick 94 | Tick  (*R. microplus -* OM760995)** [50] | Sakon Nakhon | Negative* | 33.636 | 74.64 |  |
| Tick 95 | Tick  (*Haemaphysalis* *bispinosa –* OM760853) ** [50] | Sakon Nakhon | Negative | 37.439 | 74.442 |  |
| Tick 96 | Tick  (*R. microplus* – OM761033) ** [50] | Sakon Nakhon | Negative | Undetected | 85.694 |  |
| Tick 97 | Tick  (*R. microplus*) | Sakon Nakhon | Negative | Undetected | 81.746 |  |
| Tick 98 | Tick  (*R. microplus*) | Sakon Nakhon | Negative | Undetected | 74.64 |  |
| Tick 99 | Tick  (*R. microplus* - OM760992) ** [50] | Khon Kean | Negative | Undetected | 74.837 |  |
| Tick 100 | Tick  (*R. microplus* - OM760996) ** [50] | Khon Kean | Negative | Undetected | 84.805 |  |
| Tick 101 | Tick  (*R. microplus -*OM761021) ** [50] | Loei | Negative* | 33.088 | 75.974 |  |
| Tick 102 | Tick  (*R. microplus -* OM761032)** [50] | Loei | Negative* | 33.568 | 74.983 |  |
| Tick 103 | Tick  (*R. microplus* - OM761037) ** [50] | Loei | Negative | Undetected | 82.213 |  |
| Tick 104 | Tick  (*R. microplus* - OM761042) ** [50] | Loei | Negative | Undetected | 62.504 |  |
| Tick 105 | Tick  (*R. microplus* - OM761043) ** [50] | Loei | Negative | Undetected | 75.082 |  |
| Tick 106 | Tick  (*R. microplus* - OM761038)** [50] | Bueng Kan | Negative | Undetected | 74.983 |  |
| Tick 107 | Tick  (*R. microplus* - OM761039) ** [50] | Bueng Kan | Negative* | 32.261 | 74.785 |  |
| Tick 108 | Tick  (*R. microplus* - OM761041) ** [50] | Bueng Kan | Negative | 35.486 | 74.587 |  |
| Tick 109 | Tick  (*R. microplus* - OM761033) ** [50] | Bueng Kan | Negative* | 15.795 | 85.481 |  |
| Tick 110 | Tick  (*R. microplus* - OM761024) ** [50] | Bueng Kan | Negative | 36.485 | 74.64 |  |
| Tick 111 | Tick  (*R. microplus*) | Bueng Kan | Negative | 36.447 | 74.837 |  |
| Tick 112 | Tick  (*R. microplus*) | Bueng Kan | Negative* | 32.471 | 74.837 |  |
| Tick 113 | Tick  (*R. microplus*) | Bueng Kan | Negative | Undetected | 74.936 |  |
| Tick 114 | Tick  (*R. microplus* - OM761044) ** [50] | Loei | *T. sinensis*  (PP188662) ** [51] | 34.225 | 75.725 |  |
| Tick 115 | Tick  (*R. microplus* - OM761045) ** [50] | Loei | Negative* | 33.114 | 75.429 |  |
| Tick 116 | Tick  (*R. microplus* - OM761046) ** [50] | Loei | *T. sinensis*  (PP188647) ** [51] | 33.527 | 75.923 |  |
| Tick 117 | Tick  (*R. microplus* - OM761047) ** [50] | Loei | *T. sinensis*  (PP188648) ** [51] | 34.439 | 76.022 |  |
| Tick 118 | Tick  (*R. microplus* - OM761048) ** [50] | Loei | *T. sinensis*  (PP188649) ** [51] | 33.657 | 76.022 |  |
| Tick 119 | Tick  (*R. microplus* - OM761049) ** [50] | Loei | Negative | 36.858 | 75.923 |  |
| Tick120 | Tick  (*R. microplus* - OM761050) ** [50] | Loei | *T. sinensis*  (PP188663) ** [51] | 33.518 | 75.921 |  |
| Tick121 | Tick  (*R. microplus* - OM761062) ** [50] | Loei | Negative | 35.342 | 75.921 |  |
| Tick122 | Tick  (*R. microplus* - OM761064) ** [50] | Loei | *T. sinensis*  (PP188650) ** [51] | 31.978 | 75.724 |  |
| Tick123 | Tick  (*R. microplus*) | Loei | *T. sinensis*  (PP188651) ** [51] | 32.808 | 75.823 |  |
| Tick124 | Tick  (*R. microplus*) | Loei | *T. sinensis*  (PP188652) ** [51] | 32.935 | 75.823 |  |
| Tick125 | Tick  (*R. microplus*) | Loei | *T. sinensis*  (PP188653) ** [51] | 30.114 | 75.899 |  |
| Tick150 | Tick  (*R. microplus –* OM760993)** [50] | Roi-Et | *T. sinensis* | 32.856 | 74.716 |  |
| Tick171 | Tick  (*R. microplus*) | Loei | *T. sinensis*  (PP188661) ** [51] | 31.052 | 75.871 |  |
| Tick172 | Tick  (*R. microplus*) | Loei | *T. sinensis*  (PP188655) ** [51] | 31.805 | 75.97 |  |
| Tick173 | Tick  (*R. microplus -* OM761051)** [50] | Maha Sarakham | Negative | 39.456 | 74.682 |  |
| Tick174 | Tick  (*R. microplus -* OM761052)** [50] | Nong Bua Lamphu | Negative | 37.704 | 74.781 |  |
| Tick175 | Tick  (*R. microplus*) | Nong Bua Lamphu | Negative | Undetected | 74.682 |  |
| Tick176 | Tick  (*R. microplus -* OM761053)** [50] | Nong Khai | Negative | Undetected | 75.772 | cPCR positive for *B. bigemina* |
| Tick177 | Tick  (*R. microplus* - OM761054) ** [50] | Nong Khai | Negative | Undetected | 75.772 |  |
| Tick178 | Tick  (*R. microplus* - OM761055) ** [50] | Nong Khai | Negative* | 28.982 | 74.484 | cPCR positive for *B. bigemina* |
| Tick179 | Tick  (*R. microplus* - OM761056) ** [50] | Nong Khai | Negative* | 30.736 | 75.871 | cPCR positive for *B. bigemina* |
| Tick180 | Tick  (*R. microplus* - OM761057) ** [50] | Nong Khai | Negative* | 30.311 | 75.871 |  |
| Tick181 | Tick  (*R. microplus* - OM761058) ** [50] | Nong Khai | Negative | Undetected | 74.583 | cPCR positive for *B. bigemina* |
| Tick182 | Tick  (*R. microplus* - OM761059) ** [50] | Nong Khai | *T. orientalis* | 28.295 | 74.682 |  |
| Tick184 | Tick  (*R. microplus*) | Nong Khai | Negative | Undetected | 74.88 |  |
| Tick185 | Tick  (*R. microplus*) | Nong Khai | Negative* | 26.687 | 74.781 |  |
| Tick186 | Tick  (*R. microplus*) | Nong Khai | *T. orientalis* | 26.069 | 74.682 |  |
| Tick187 | Tick  (*R. microplus*) | Nong Khai | *T. orientalis* | 32.388 | 74.286 |  |
| Tick188 | Tick  (*R. microplus*) | Nong Khai | *T. orientalis* | 30.359 | 74.583 |  |
| Tick189 | Tick  (*R. microplus*) | Nong Khai | Negative | Undetected | 74.682 |  |
| Tick190 | Tick  (*R. microplus*) | Nong Khai | Negative | 38.995 | 74.781 |  |
| Tick191 | Tick  (*R. microplus*) | Nong Khai | Negative | Undetected | 75.871 |  |
| Tick192 | Tick  (*R. microplus*) | Nong Khai | Negative | Undetected | 84.782 |  |
| Tick193 | Tick  (*Haemaphysalis bispinosa -* OM760846)** [50] | Nakhon Phanom | Negative | Undetected | 74.88 |  |
| Tick194 | Tick  (*Haemaphysalis bispinosa -* OM760847)** [50] | Nakhon Phanom | Negative | 36.652 | 74.682 |  |
| Tick195 | Tick  (*Haemaphysalis bispinosa -* OM760848)** [50] | Nakhon Phanom | Negative* | 28.498 | 74.682 |  |
| Tick196 | Tick  (*Haemaphysalis bispinosa -* OM760849)** [50] | Nakhon Phanom | Negative | 36.475 | 74.88 |  |
| Tick197 | Tick  (*R. microplus*- OM761015) ** [50] | Nakhon Phanom | Negative | Undetected | 60.424 |  |
| Tick198 | Tick  (*R. microplus* - OM761016) ** [50] | Nakhon Phanom | Negative | 39.515 | 74.88 |  |

*: The cycle number real-time PCR was less than the cutoff (<35 cycles), but Tm does not match any positive control of *Theileria* species.

**: Representative GenBank accession numbers of the submitted sequences.
