# Supplementary material for: Development and validation of a real-time SYBR green PCR method for the detection and differentiation of Babesia and Theileria species (Apicomplexa: Piroplasmida) in hard ticks and cattle blood from Thailand
Source: Parasite. 2025 Aug 25;32:54. doi: 10.1051/parasite/2025040 (PMC12380414; doi:10.1051/parasite/2025040)
Supplement: Supplementary file 1 — Supplementary Figure 1: Homology comparisons of reference sequences and sequences submitted in this study for tick species identification. OR545517: reference sequence of R. microplus, OR335052: reference sequence of H. bispinosa, Representative sequences from each province (KK: Khon Kaen, BK: Bueng Kan, SK: Sakon Nakhon, NP: Nakhon Phanom, LI: Loei, RE: Roi Et, and MS: Maha Sarakham), were aligned. Supplementary Figure 2: Comparison of plasmid control sequences with GenBank references. a) Plasmid controls: B. bigemina (PV751017) and B. bovis (PV751018), reference sequences: B. bigemina (OP361312) and B. bovis (CP125253), b) Plasmid controls: T. annulata (PV751019), T. orientalis (PV774666), and T. sinensis (PV774665), references sequences: T. annulata (MT341858), T. orientalis (MH208642), and T. sinensis (MT271911). Table S1: Real-Time PCR results of Theileria detection. Table S2: Real-Time PCR results of Babesia detection. [file parasite-32-54-s1.zip › Table S2 clean.docx]

**Table S2.** Real-Time PCR result of *Babesia* detection.

| Sample name | Sample type | Location | Result (cPCR) | Result (qPCR) C_t_ (cutoff<35) | T_m_ | Remark |
| --- | --- | --- | --- | --- | --- | --- |
| MKM194 | cow blood | Maha Sarakham | Negative | 38.015 | 75.031 |  |
| MKM195 | cow blood | Maha Sarakham | Negative | Undetected | 75.031 |  |
| MKM196 | cow blood | Maha Sarakham | Negative | Undetected | 61.312 |  |
| MKM197 | cow blood | Maha Sarakham | Negative | 38.96 | 75.328 |  |
| MKM198 | cow blood | Maha Sarakham | Negative | Undetected | 73.748 |  |
| MKM199 | cow blood | Maha Sarakham | Negative | Undetected | 63.779 |  |
| MKM200 | cow blood | Maha Sarakham | Negative | Undetected | 64.964 |  |
| MKM201 | cow blood | Maha Sarakham | Negative | Undetected | 93.983 |  |
| MKM202 | cow blood | Maha Sarakham | Negative | Undetected | 75.328 |  |
| MKM203 | cow blood | Maha Sarakham | Negative | Undetected | 61.015 |  |
| MKM204 | cow blood | Maha Sarakham | Negative | Undetected | 61.114 |  |
| MKM205 | cow blood | Maha Sarakham | Negative | Undetected | 62.595 |  |
| MKM206 | cow blood | Maha Sarakham | Negative | Undetected | 73.551 |  |
| MKM207 | cow blood | Maha Sarakham | Negative | Undetected | 77.894 |  |
| MKM208 | cow blood | Maha Sarakham | Negative | Undetected | 74.834 |  |
| MKM209 | cow blood | Maha Sarakham | Negative | 38.566 | 74.735 |  |
| MKM210 | cow blood | Maha Sarakham | Negative | Undetected | 75.13 |  |
| MKM211 | cow blood | Maha Sarakham | Negative | Undetected | 61.015 |  |
| MKM212 | cow blood | Maha Sarakham | Negative | Undetected | 75.229 |  |
| MKM213 | cow blood | Maha Sarakham | Negative | Undetected | 63.384 |  |
| MKM214 | cow blood | Maha Sarakham | Negative | Undetected | 61.213 |  |
| MKM215 | cow blood | Maha Sarakham | Negative | 39.568 | 74.834 |  |
| MKM216 | cow blood | Maha Sarakham | Negative | 38.854 | 74.933 |  |
| MKM217 | cow blood | Maha Sarakham | Negative | Undetected | 62.002 |  |
| MKM218 | cow blood | Maha Sarakham | Negative | Undetected | 60.621 |  |
| MKM219 | cow blood | Maha Sarakham | Negative | Undetected | 78.289 |  |
| MKM220 | cow blood | Maha Sarakham | Negative | Undetected | 61.509 |  |
| MKM221 | cow blood | Maha Sarakham | Negative | Undetected | 62.299 |  |
| MKM222 | cow blood | Maha Sarakham | Negative | 39.269 | 75.031 |  |
| MKM223 | cow blood | Maha Sarakham | Negative | Undetected | 75.13 |  |
| MKM224 | cow blood | Maha Sarakham | Negative | Undetected | 83.224 |  |
| MKM225 | cow blood | Maha Sarakham | Negative | Undetected | 80.756 |  |
| KKN 2 | cow blood | Khon Kean | Negative | Undetected | 75.13 |  |
| KKN 3 | cow blood | Khon Kean | Negative | Undetected | 75.13 |  |
| KKN 4 | cow blood | Khon Kean | Negative | Undetected | 61.015 |  |
| KKN 5 | cow blood | Khon Kean | Negative | Undetected | 61.312 |  |
| KKN 6 | cow blood | Khon Kean | Negative | Undetected | 72.959 |  |
| KKN 7 | cow blood | Khon Kean | Negative | Undetected | 80.164 |  |
| KKN 8 | cow blood | Khon Kean | Negative | Undetected | 75.328 |  |
| KKN 9 | cow blood | Khon Kean | Negative | Undetected | 93.193 |  |
| KKN 10 | cow blood | Khon Kean | Negative | Undetected | 91.811 |  |
| KKN 11 | cow blood | Khon Kean | Negative | Undetected | 75.031 |  |
| KKN 12 | cow blood | Khon Kean | Negative | Undetected | 75.328 |  |
| KKN 13 | cow blood | Khon Kean | Negative | Undetected | 75.426 |  |
| KKN 14 | cow blood | Khon Kean | Negative | Undetected | 74.933 |  |
| KKN 15 | cow blood | Khon Kean | Negative | Undetected | 81.546 |  |
| KKN 16 | cow blood | Khon Kean | Negative | Undetected | 75.13 |  |
| KKN 17 | cow blood | Khon Kean | Negative | Undetected | 75.229 |  |
| KKN 18 | cow blood | Khon Kean | Negative | Undetected | 90.627 |  |
| KKN 19 | cow blood | Khon Kean | Negative | Undetected | 81.349 |  |
| KKN 20 | cow blood | Khon Kean | Negative | Undetected | 75.328 |  |
| KKN 21 | cow blood | Khon Kean | Negative | Undetected | 88.455 |  |
| KKN 22 | cow blood | Khon Kean | Negative | Undetected | 60.522 |  |
| KKN 23 | cow blood | Khon Kean | Negative | Undetected | 75.031 |  |
| NMA 8 | cow blood | Nakhon Ratchasima | *T. sinensis*  (PV592339) ** | Undetected | 93.672 |  |
| NMA 83 | cow blood | Nakhon Ratchasima | *T. orientalis*  (PV592336) ** | Undetected | 93.672 |  |
| NMA 88 | cow blood | Nakhon Ratchasima | *T. orientalis*  (PV592336) ** | Undetected | 93.672 |  |
| NMA128 | cow blood | Nakhon Ratchasima | Negative | Undetected | 68.908 |  |
| NMA129 | cow blood | Nakhon Ratchasima | Negative | 38.863 | 74.433 |  |
| NMA130 | cow blood | Nakhon Ratchasima | Negative | Undetected | 74.137 |  |
| NMA131 | cow blood | Nakhon Ratchasima | Negative | Undetected | 91.896 |  |
| NMA132 | cow blood | Nakhon Ratchasima | Negative | Undetected | 74.63 |  |
| NMA133 | cow blood | Nakhon Ratchasima | Negative | Undetected | 74.433 |  |
| NMA134 | cow blood | Nakhon Ratchasima | Negative | Undetected | 93.672 |  |
| NMA135 | cow blood | Nakhon Ratchasima | Negative | Undetected | 72.164 |  |
| NMA136 | cow blood | Nakhon Ratchasima | Negative | Undetected | 74.433 |  |
| NMA137 | cow blood | Nakhon Ratchasima | Negative | Undetected | 81.931 |  |
| NMA139 | cow blood | Nakhon Ratchasima | Negative | Undetected | 74.236 |  |
| NMA138 | cow blood | Nakhon Ratchasima | Negative | Undetected | 85.582 |  |
| NMA141 | cow blood | Nakhon Ratchasima | Negative | Undetected | 61.114 |  |
| NMA140 | cow blood | Nakhon Ratchasima | Negative | Undetected | 81.438 |  |
| NMA143 | cow blood | Nakhon Ratchasima | Negative | Undetected | 92.291 |  |
| NMA142 | cow blood | Nakhon Ratchasima | Negative | Undetected | 61.311 |  |
| NMA146 | cow blood | Nakhon Ratchasima | Negative | Undetected | 91.107 |  |
| NMA144 | cow blood | Nakhon Ratchasima | Negative | Undetected | 86.371 |  |
| NMA147 | cow blood | Nakhon Ratchasima | Negative | Undetected | 91.403 |  |
| NMA148 | cow blood | Nakhon Ratchasima | Negative | Undetected | 93.277 |  |
| NMA149 | cow blood | Nakhon Ratchasima | Negative | Undetected | 60.818 |  |
| NMA150 | cow blood | Nakhon Ratchasima | Negative | Undetected | 61.213 |  |
| NMA151 | cow blood | Nakhon Ratchasima | Negative | Undetected | 74.433 |  |
| NMA152 | cow blood | Nakhon Ratchasima | Negative | Undetected | 61.805 |  |
| NMA153 | cow blood | Nakhon Ratchasima | Negative | Undetected | 74.236 |  |
| NMA154 | cow blood | Nakhon Ratchasima | Negative | Undetected | 60.522 |  |
| NMA155 | cow blood | Nakhon Ratchasima | Negative | Undetected | 62.396 |  |
| NMA156 | cow blood | Nakhon Ratchasima | Negative | Undetected | 74.433 |  |
| NMA157 | cow blood | Nakhon Ratchasima | Negative | Undetected | 62.89 |  |
| NMA158 | cow blood | Nakhon Ratchasima | Negative | Undetected | 64.172 |  |
| NMA159 | cow blood | Nakhon Ratchasima | Negative | Undetected | 78.084 |  |
| NMA160 | cow blood | Nakhon Ratchasima | Negative | Undetected | 74.532 |  |
| NMA174 | cow blood | Nakhon Ratchasima | *T. orientalis*  (PV592335) ** | Undetected | 74.206 |  |
| NST1 | cow blood | Nakhon Si Thammarat | *B. bigemina* | 35.656 | 74.334 | Mix *T. sinensis* |
| NST2 | cow blood | Nakhon Si Thammarat | *T. sinensis* (PV592343) ** | Undetected | 85.779 |  |
| NST3 | cow blood | Nakhon Si Thammarat | *B. bigemina* | Undetected | 61.409 |  |
| NST4 | cow blood | Nakhon Si Thammarat | *T. sinensis*  (PV592339) ** | Undetected | 82.523 |  |
| NST5 | cow blood | Nakhon Si Thammarat | *T. sinensis*  (PV592339) ** | 38.748 | 74.63 |  |
| NST6 | cow blood | Nakhon Si Thammarat | *T. sinensis*  (PV592339) ** | Undetected | 74.532 |  |
| NST7 | cow blood | Nakhon Si Thammarat | *T. sinensis*  (PV592340) ** | 38.757 | 74.63 |  |
| NST8 | cow blood | Nakhon Si Thammarat | *T. sinensis*  (PV592339) ** | 38.757 | 74.63 |  |
| NST9 | cow blood | Nakhon Si Thammarat | *T. sinensis*  (PV592339) ** | Undetected | 61.41 |  |
| NST10 | cow blood | Nakhon Si Thammarat | Negative | Undetected | 74.532 |  |
| NST11 | cow blood | Nakhon Si Thammarat | *T. sinensis*  (PV592339) ** | Undetected | 74.334 |  |
| NST12 | cow blood | Nakhon Si Thammarat | Negative | Undetected | 85.68 |  |
| NST14 | cow blood | Nakhon Si Thammarat | *T. sinensis*  (PV592341) ** | Undetected | 85.68 |  |
| NST15 | cow blood | Nakhon Si Thammarat | *T. sinensis*  (PV592339) ** | Undetected | 85.68 |  |
| NST16 | cow blood | Nakhon Si Thammarat | *B. bigemina* | Undetected | 74.503 | Mix *T. sinensis* |
| NST17 | cow blood | Nakhon Si Thammarat | *T. sinensis*  (PV592339) ** | Undetected | 85.68 |  |
| NST19 | cow blood | Nakhon Si Thammarat | *T. sinensis*  (PV592342) ** | Undetected | 85.68 |  |
| NST20 | cow blood | Nakhon Si Thammarat | *T. sinensis*  (PV592339) ** | 36.829 | 74.433 |  |
| NST21 | cow blood | Nakhon Si Thammarat | Negative | Undetected | 74.63 |  |
| NST22 | cow blood | Nakhon Si Thammarat | Negative | Undetected | 74.63 |  |
| NST23 | cow blood | Nakhon Si Thammarat | Negative | 33.237* | 74.532 |  |
| NST24 | cow blood | Nakhon Si Thammarat | Negative | Undetected | 61.4 |  |
| NST25 | cow blood | Nakhon Si Thammarat | Negative | Undetected | 75.824 |  |
| NST26 | cow blood | Nakhon Si Thammarat | Negative | Undetected | 74.63 |  |
| NST27 | cow blood | Nakhon Si Thammarat | Negative | 37.761 | 74.433 |  |
| NST28 | cow blood | Nakhon Si Thammarat | Negative | Undetected | 74.63 |  |
| NST29 | cow blood | Nakhon Si Thammarat | Negative | Undetected | 74.602 |  |
| NST30 | cow blood | Nakhon Si Thammarat | *B. bigemina* | 38.462 | 74.503 | Mix *T. sinensis* |
| NST31 | cow blood | Nakhon Si Thammarat | Negative | Undetected | 80.846 |  |
| NST32 | cow blood | Nakhon Si Thammarat | Negative | Undetected | 82.425 |  |
| NST33 | cow blood | Nakhon Si Thammarat | Negative | Undetected | 83.017 |  |
| NST34 | cow blood | Nakhon Si Thammarat | Negative | Undetected | 85.779 |  |
| NST35 | cow blood | Nakhon Si Thammarat | Negative | 38.163 | 74.63 |  |
| NST36 | cow blood | Nakhon Si Thammarat | Negative | Undetected | 74.334 |  |
| NST37 | cow blood | Nakhon Si Thammarat | Negative | Undetected | 82.326 |  |
| NST38 | cow blood | Nakhon Si Thammarat | Negative | Undetected | 74.334 |  |
| NST39 | cow blood | Nakhon Si Thammarat | Negative | Undetected | 74.532 |  |
| NST40 | cow blood | Nakhon Si Thammarat | Negative | Undetected | 74.63 |  |
| NST41 | cow blood | Nakhon Si Thammarat | Negative | Undetected | 85.68 |  |
| NST42 | cow blood | Nakhon Si Thammarat | Negative | Undetected | 61.114 |  |
| NST43 | cow blood | Nakhon Si Thammarat | Negative | Undetected | 74.63 |  |
| NST43 | cow blood | Nakhon Si Thammarat | Negative | Undetected | 68.704 |  |
| RET1 | cow blood | Roi-Et | Negative | Undetected | 72.524 |  |
| RET2 | cow blood | Roi-Et | Negative | Undetected | 82.043 |  |
| RET3 | cow blood | Roi-Et | Negative | Undetected | 74.11 |  |
| RET4 | cow blood | Roi-Et | Negative | Undetected | 74.408 |  |
| RET5 | cow blood | Roi-Et | Negative | Undetected | 72.326 |  |
| RET6 | cow blood | Roi-Et | Negative | Undetected | 74.408 |  |
| RET7 | cow blood | Roi-Et | Negative | Undetected | 85.513 |  |
| RET8 | cow blood | Roi-Et | Negative | Undetected | 72.425 |  |
| RET9 | cow blood | Roi-Et | Negative | Undetected | 74.309 |  |
| RET10 | cow blood | Roi-Et | Negative | Undetected | 74.408 |  |
| RET11 | cow blood | Roi-Et | Negative | Undetected | 74.21 |  |
| Tick 22 | Tick  (*R. microplus –* OM760994)** [50] | Mukdahan | *Anaplasma* | Undetected | 85.593 |  |
| Tick 67 | Tick  (*R. microplus -* OM761040)** [50] | Bueng Kan | *Anaplasma* | Undetected | 85.205 |  |
| Tick 81 | Tick  (*R. microplus –* OM761006)** [50] | Nakhon Phanom | *T. orientalis*  (PP330060) ** [51] | Undetected | 85.602 |  |
| Tick 92 | Tick  (*R.* *microplus –* OM760991)** [50] | Khon Kean | Negative | Undetected | 60.917 |  |
| Tick 93 | Tick  (*R. microplus -*OM761020)** [50] | Loei | Negative | Undetected | 91.318 |  |
| Tick 94 | Tick  (*R. microplus -* OM760995)** [50] | Sakon Nakhon | Negative | Undetected | 85.297 |  |
| Tick 95 | Tick  (*Haemaphysalis* *bispinosa –* OM760853 ** [50] | Sakon Nakhon | Negative | Undetected | 75.13 |  |
| Tick 96 | Tick  (*R. microplus –* OM761033) ** [50] | Sakon Nakhon | Negative | Undetected | 83.026 |  |
| Tick 97 | Tick  (*R. microplus*) | Sakon Nakhon | Negative | Undetected | 82.533 |  |
| Tick 98 | Tick  (*R. microplus*) | Sakon Nakhon | Negative | Undetected | 85.593 |  |
| Tick 99 | Tick  (*R. microplus -* OM760992) ** [50] | Khon Kean | Negative | Undetected | 82.434 |  |
| Tick 100 | Tick  (*R. microplus -* OM760996) ** [50] | Khon Kean | Negative | Undetected | 85.79 |  |
| Tick 101 | Tick  (*R. microplus -* OM761021) ** [50] | Loei | Negative | Undetected | 82.138 |  |
| Tick 102 | Tick  (*R. microplus -*OM761032) ** [50] | Loei | Negative | Undetected | 85.395 |  |
| Tick 103 | Tick  (*R. microplus -* OM761037) ** [50] | Loei | Negative | Undetected | 61.706 |  |
| Tick 104 | Tick  (*R. microplus -*OM761042) ** [50] | Loei | Negative | Undetected | 82.533 |  |
| Tick 105 | Tick  (*R. microplus -* OM761043) ** [50] | Loei | Negative | Undetected | 82.434 |  |
| Tick 106 | Tick  (*R. microplus -* OM761038) ** [50] | Bueng Kan | Negative | Undetected | 85.593 |  |
| Tick 107 | Tick  (*R. microplus -*OM761039) ** [50] | Bueng Kan | Negative | Undetected | 85.79 |  |
| Tick 108 | Tick  (*R. microplus -* OM761041) ** [50] | Bueng Kan | Negative | Undetected | 85.889 |  |
| Tick 109 | Tick  (*R. microplus -*OM761033) ** [50] | Bueng Kan | Negative | Undetected | 82.039 |  |
| Tick 110 | Tick  (*R. microplus-* OM761024) ** [50] | Bueng Kan | Negative | Undetected | 61.312 |  |
| Tick 111 | Tick  (*R. microplus*) | Bueng Kan | Negative | Undetected | 75.031 |  |
| Tick 112 | Tick  (*R. microplus*) | Bueng Kan | Negative | Undetected | 74.735 |  |
| Tick 113 | Tick  (*R. microplus*) | Bueng Kan | Negative | Undetected | 75.426 |  |
| Tick 114 | Tick  (*R. microplus* -OM761044) ** [50] | Loei | *T. sinensis*  (PP188662) ** [51] | Undetected | 73.946 |  |
| Tick 115 | Tick  (*R. microplus -* OM761045) ** [50] | Loei | Negative | Undetected | 82.632 |  |
| Tick 116 | Tick  (*R. microplus -* OM761046) ** [50] | Loei | *T. sinensis*  (PP188647) ** [51] | Undetected | 75.13 |  |
| Tick 117 | Tick  (*R. microplus -* OM761047) ** [50] | Loei | *T. sinensis*  (PP188648) ** [51] | Undetected | 74.933 |  |
| Tick 118 | Tick  (*R. microplus -* OM761048) ** [50] | Loei | *T. sinensis*  (PP188649) ** [51] | 39.833 | 74.439 |  |
| Tick 119 | Tick  (*R. microplus -* OM761049) ** [50] | Loei | Negative | Undetected | 75.13 |  |
| Tick120 | Tick  (*R. microplus -* OM761050) ** [50] | Loei | *T. sinensis*  (PP188663) ** [51] | Undetected | 74.637 |  |
| Tick121 | Tick  (*R. microplus -* OM761062) ** [50] | Loei | Negative | Undetected | 75.229 |  |
| Tick122 | Tick  (*R. microplus -* OM761064) ** [50] | Loei | *T. sinensis*  (PP188650) ** [51] | Undetected | 73.946 |  |
| Tick123 | Tick  (*R. microplus*) | Loei | *T. sinensis*  (PP188651) ** [51] | 35.094 | 75.535 |  |
| Tick124 | Tick  (*R. microplus*) | Loei | *T. sinensis*  (PP188652) ** [51] | 35.020 | 75.535 |  |
| Tick125 | Tick  (*R. microplus*) | Loei | *T. sinensis*  (PP188653) ** [51] | Undetermined | 66.252 |  |
| Tick150 | Tick  (*R. microplus –* OM760993)** [50] | Roi-Et | *T. sinensis* | 32.509* | 75.041 |  |
| Tick171 | Tick  (*R. microplus*) | Loei | *T. sinensis*  (PP188661) ** [51] | 34.287* | 75.140 |  |
| Tick172 | Tick  (*R. microplus*) | Loei | *T. sinensis*  (PP188665) ** [51] | Undetermined | 83.732 |  |
| Tick173 | Tick  (*R. microplus –* OM761051)** [50] | Maha Sarakham | Negative | Undetermined | 61.116 |  |
| Tick174 | Tick  (*R. microplus -* OM761052)** [50] | Nong Bua Lamphu | Negative | Undetermined | 83.732 |  |
| Tick175 | Tick  (*R. microplus*) | Nong Bua Lamphu | Negative | Undetermined | 73.066 |  |
| Tick176 | Tick  (*R. microplus –* OM761053)** [50] | Nong Khai | *B. bigemina* | 26.614 | 74.385 |  |
| Tick177 | Tick  (*R. microplus -* OM761054) ** [50] | Nong Khai | Negative | 32.304* | 75.239 |  |
| Tick178 | Tick  (*R. microplus -* OM761055) ** [50] | Nong Khai | *B. bigemina* | 33.649 | 74.418 |  |
| Tick179 | Tick  (*R. microplus -* OM761056) ** [50] | Nong Khai | *B. bigemina* | 32.786 | 74.372 |  |
| Tick180 | Tick  (*R. microplus -* OM761057) ** [50] | Nong Khai | Negative | Undetermined | 79.979 |  |
| Tick181 | Tick  (*R. microplus -* OM761058) ** [50] | Nong Khai | *B. bigemina* | 34.063 | 74.375 |  |
| Tick182 | Tick  (*R. microplus -* OM761059) ** [50] | Nong Khai | *T. orientalis* | 34.569* | 75.634 |  |
| Tick184 | Tick  (*R. microplus*) | Nong Khai | Negative | Undetermined | 64.869 |  |
| Tick185 | Tick  (*R. microplus*) | Nong Khai | Negative | 30.288* | 75.041 |  |
| Tick186 | Tick  (*R. microplus*) | Nong Khai | *T. orientalis* | 31.714* | 75.041 |  |
| Tick187 | Tick  (*R. microplus*) | Nong Khai | *T. orientalis* | Undetermined | 74.943 |  |
| Tick188 | Tick  (*R. microplus*) | Nong Khai | *T. orientalis* | Undetermined | 86.103 |  |
| Tick189 | Tick  (*R. microplus*) | Nong Khai | Negative | Undetermined | 61.313 |  |
| Tick190 | Tick  (*R. microplus*) | Nong Khai | Negative | Undetermined | 61.017 |  |
| Tick191 | Tick  (*R. microplus*) | Nong Khai | Negative | Undetermined | 67.733 |  |
| Tick192 | Tick  (*R. microplus*) | Nong Khai | Negative | Undetermined | 85.905 |  |
| Tick193 | Tick  (*Haemaphysalis bispinosa -* OM760846) ** [50] | Nakhon Phanom | Negative | Undetermined | 60.918 |  |
| Tick194 | Tick  (*Haemaphysalis bispinosa -* OM760847) ** [50] | Nakhon Phanom | Negative | Undetermined | 82.053 |  |
| Tick195 | Tick  (*Haemaphysalis* *bispinosa -* OM760848) ** [50] | Nakhon Phanom | Negative | Undetermined | 85.806 |  |
| Tick196 | Tick  (*Haemaphysalis bispinosa -* OM760849) ** [50] | Nakhon Phanom | Negative | Undetermined | 76.226 |  |
| Tick197 | Tick  (*R. microplus -* OM761015) ** [50] | Nakhon Phanom | Negative | 32.487* | 62.301 |  |
| Tick198 | Tick  (*R. microplus -* OM761016) ** [50] | Nakhon Phanom | Negative | 34.729* | 75.733 |  |

*: The cycle number real-time PCR was less than the cutoff (<35 cycles), but Tm does not match any positive control of *Babesia* species.

**: Representative GenBank accession numbers of the submitted sequences.
