# Supplementary figures and images for: Development and validation of a real-time SYBR green PCR method for the detection and differentiation of Babesia and Theileria species (Apicomplexa: Piroplasmida) in hard ticks and cattle blood from Thailand
Source: Parasite. 2025 Aug 25;32:54. doi: 10.1051/parasite/2025040 (PMC12380414; doi:10.1051/parasite/2025040)

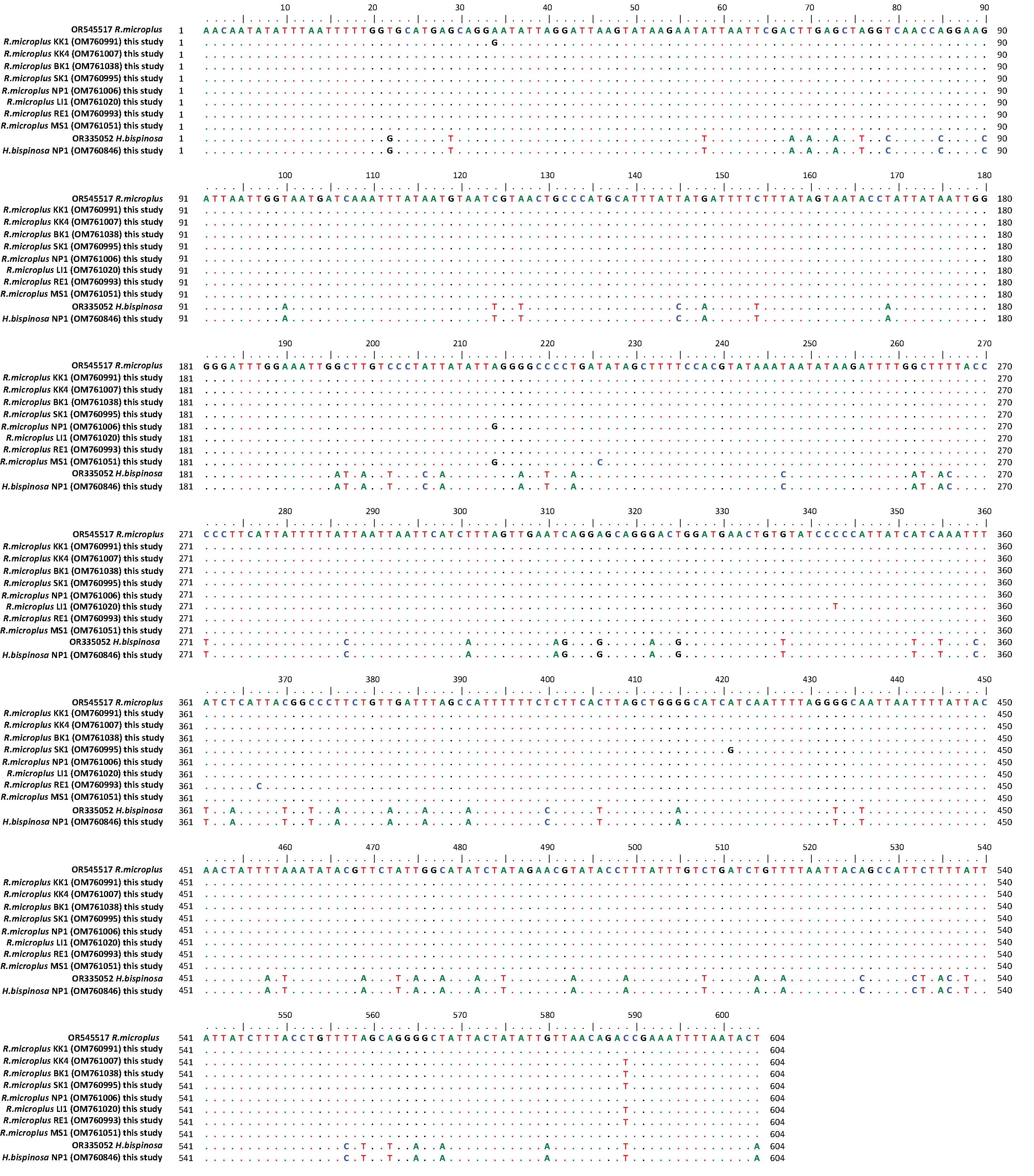

Supplement: Supplementary file 1 — Supplementary Figure 1: Homology comparisons of reference sequences and sequences submitted in this study for tick species identification. OR545517: reference sequence of R. microplus, OR335052: reference sequence of H. bispinosa, Representative sequences from each province (KK: Khon Kaen, BK: Bueng Kan, SK: Sakon Nakhon, NP: Nakhon Phanom, LI: Loei, RE: Roi Et, and MS: Maha Sarakham), were aligned. Supplementary Figure 2: Comparison of plasmid control sequences with GenBank references. a) Plasmid controls: B. bigemina (PV751017) and B. bovis (PV751018), reference sequences: B. bigemina (OP361312) and B. bovis (CP125253), b) Plasmid controls: T. annulata (PV751019), T. orientalis (PV774666), and T. sinensis (PV774665), references sequences: T. annulata (MT341858), T. orientalis (MH208642), and T. sinensis (MT271911). Table S1: Real-Time PCR results of Theileria detection. Table S2: Real-Time PCR results of Babesia detection. [file parasite-32-54-s1.zip › Revised 2 SF1.tif]

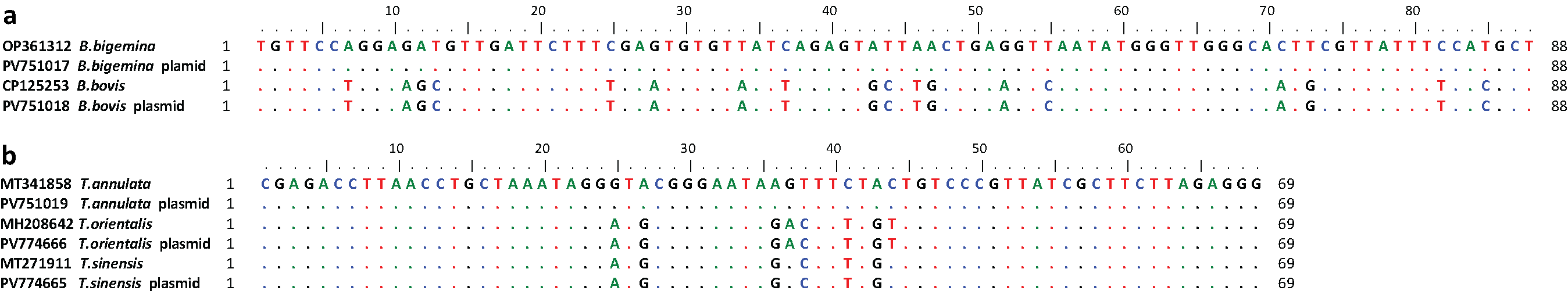

Supplement: Supplementary file 1 — Supplementary Figure 1: Homology comparisons of reference sequences and sequences submitted in this study for tick species identification. OR545517: reference sequence of R. microplus, OR335052: reference sequence of H. bispinosa, Representative sequences from each province (KK: Khon Kaen, BK: Bueng Kan, SK: Sakon Nakhon, NP: Nakhon Phanom, LI: Loei, RE: Roi Et, and MS: Maha Sarakham), were aligned. Supplementary Figure 2: Comparison of plasmid control sequences with GenBank references. a) Plasmid controls: B. bigemina (PV751017) and B. bovis (PV751018), reference sequences: B. bigemina (OP361312) and B. bovis (CP125253), b) Plasmid controls: T. annulata (PV751019), T. orientalis (PV774666), and T. sinensis (PV774665), references sequences: T. annulata (MT341858), T. orientalis (MH208642), and T. sinensis (MT271911). Table S1: Real-Time PCR results of Theileria detection. Table S2: Real-Time PCR results of Babesia detection. [file parasite-32-54-s1.zip › Revised SF2_R3.tif]
